# Neural basis of the attention bias during addiction stroop task in methamphetamine-dependent patients with and without a history of psychosis: an ERP study

**DOI:** 10.3389/fpsyg.2023.1173711

**Published:** 2023-06-09

**Authors:** Gengdi Huang, Chuanliang Han, Jihui Yang, Caihong Ye, Iqbal Javed, Fen Liu, Zhi Kong, Ying Li, Yingmei Zhu, Guangyong Yi, Chuanjia Ju, Xiaojian Jia, Mei Yang

**Affiliations:** ^1^State Key Laboratory of Chemical Oncogenomics, Guangdong Provincial Key Laboratory of Chemical Genomics, Peking University Shenzhen Graduate School, Shenzhen, China; ^2^Department of Addiction Medicine, Shenzhen Clinical Research Center for Mental Disorders, Shenzhen Kangning Hospital, Shenzhen Mental Health Center, the School of Mental Health of Southern University of Science and Technology, Shenzhen University, Anhui Medical University, and Jining Medical College, Shenzhen, China; ^3^Shenzhen Key Laboratory of Neuropsychiatric Modulation and Collaborative Innovation Center for Brain Science, Guangdong Provincial Key Laboratory of Brain Connectome and Behavior, CAS Center for Excellence in Brain Science and Intelligence Technology, Brain Cognition and Brain Disease Institute, Shenzhen Institute of Advanced Technology, Chinese Academy of Sciences, Shenzhen-Hong Kong Institute of Brain Science, Shenzhen Fundamental Research Institutions, Shenzhen, China

**Keywords:** methamphetamine, methamphetamine-associated psychosis, addiction stroop task, attention bias, EEG

## Abstract

**Background:**

Attentional bias plays an important role in sustaining various types of drug addiction. No prior studies examined methamphetamine (MA)-associated psychosis (MAP) relationships between ERP time course and performance on an addiction Stroop task in MA abusers. The aim of the present study was to determine whether MA abusers with (MAP+) or without (MAP-) psychosis exhibit alterations of the ERP during the addiction Stroop task.

**Methods:**

Thirty-one healthy controls (CTRL), 14 MAP-, and 24 MAP+ participants were recruited and completed the addiction Stroop task during EEG recording using 32 electrodes. Group variations were compared on measures of behavioral task performance and event-related potentials (ERP) of performance monitoring (N200, P300, N450). The Barratt impulsiveness scores were analyzed to investigate correlations with ERP changes.

**Results:**

MA-related word stimulus elicited a more negative N200 amplitude over left-anterior electrodes in MAP- abusers; furthermore, a positive association between the N200 amplitude and Barratt attentional scores and non-planning scores was observed, while no such differences were found in MAP+ abusers. There were no significant differences in reaction time (RT) and error rate between each group.

**Conclusion:**

This is the first study to examine psychosis relationships between ERP time course and performance on an addiction Stroop task in MA abusers with or without psychosis. These findings support the association between attentional bias measured by the MA addiction Stroop task and N200 component as well as indicate the possibility of using this cognitive task in combination with ERP technology to detect psychosis factors among abstinent MA abusers.

## Introduction

Methamphetamine is a psychomotor stimulant with high liability for abuse, and MA abuse has become a public health concern across the globe. MA abuse has been of particular concern for a number of reasons including its association with the psychotic and cognitive symptoms that are similar to those observed in schizophrenia (Hsieh et al., 2014). Methamphetamine-associated psychosis is commonly referred to psychiatric services, displaying signs of positive symptoms such as delusions, paranoia and persecutory ideation and hallucinations (Sommers et al., 2006; Yang et al., 2020). Research has found that MAP is present in up to half of those with chronic methamphetamine dependence, leading to a heavier burden on drug treatment services (Grant, 2012). Further detection is needed to understand the underlying mechanism between MA abusers with (MAP+) or without (MAP-) psychosis.

The involvement of cognitive control modifications in addictive behaviors is evident through the presence of attentional bias, impaired decision-making, deficient response inhibition, and compulsive maladaptive behaviors (Goldstein and Volkow, 2011; Zilverstand et al., 2018). Furthermore, the cognitive processing of addiction-related stimuli is a key factor in substance cue reactivity; thus, it is essential to consider when exploring the neural basis of cognitive processing of exposures to substance cues on drug-seeking, craving, and relapse. The Stroop task (MacLeod, 1991) necessitates cognitive control as successful execution of the task demands individuals to react to one aspect of a stimulus while disregarding another contradictory element. More specifically, participants are directed to identify the ink color of a sequence of color words, consequently inhibiting the instinctive inclination to read the semantic meaning of the words. Previous research has demonstrated the association of color-naming Stroop performance in substance abuse, and MA-dependent subjects have made more errors and responded more slowly than controls in the color-naming Stroop task (Nestor et al., 2011). A related task is the addiction Stroop task (Cox et al., 2006), an analogous task of the classic color-naming Stroop task, where matched neutral and addiction-related words are used. Previous research using an addiction Stroop task has determined that the interference effects, such as an extended reaction time and increased error rate, are indicative of an attentional bias toward substance-related cues (Cox et al., 2006). Attentional bias in drug-word Stroop tests is thought to be a factor in the maintenance of drug-taking behavior and has been shown to be a reliable predictor of relapse elapse (Cox et al., 2002; Waters et al., 2003; Poireau et al., 2022). Attention bias assessed using the MA addiction Stroop task in MA abusers showed impairment in terms of a higher error rate of MA-related words relative to the CTRL participants (Chen et al., 2020). However, there is a scarcity of understanding regarding attention bias impairment in MAP+ abusers.

Event-related potentials (ERPs), high temporal resolution measures of human brain processing, have revealed the temporal sequence of the sub-processes involved in the Stroop interference and conflict resolution (Zhao et al., 2020). Previous research has suggested that a fronto-central negative-polarity effect in the 200 to 350 latency range, often referred to as the N200 (Folstein and Van Petten, 2008), can be elicited by conflict in a Stroop task (Boenke et al., 2009), and later, an N450-latency effect can also be observed (Larson et al., 2014). However, no difference was found in P300 latency or amplitude between the congruent and incongruent stimuli (Rosenfeld and Skogsberg, 2006). Electroencephalography (EEG) has been proposed as a neurophysiological biomarker to delineate psychotic disorders, expanding our understanding of the underlying neural mechanisms (Cao et al., 2022; Han et al., 2022a,b; Wang Q. et al., 2022). There has been limited research investigating patterns of the EEG that characterize MA abusers to detect electrophysiological abnormalities of their cortical networks and their associations with behavioral factors, including reduced working memory performance (Newton et al., 2003). A power spectrum analysis revealed an apparent EEG slowing in MA abusers (Newton et al., 2004). An ERP study of MA addiction Stroop task showed that attentional ERP components such as P300 were reduced with decreased craving within the first 3 abstinent months, and increased P300 amplitudes elicited by MA-related words were observed over left-anterior electrode sites (Haifeng et al., 2015). In addition, disruptions to resting EEG microstates were observed in MA abusers, leading to alterations in the microstate topographies over time, and these variations were associated with attention bias and a history of drug use (Chen et al., 2020). Recent research suggests that the left frontal electrode plays a distinct role in MAP. During resting eyes closed, MAP+ showed a higher delta/alpha frequency activity globally, while during resting eyes open, MAP+ displayed a higher delta/alpha frequency activity in all electrodes except the left frontal, when compared to the CTRL. Additionally, during the cognitive task, MAP+ exhibited a higher delta/alpha frequency activity in all electrodes except the left frontal (Howells et al., 2018). An EEG delta/alpha frequency activity assessment can help to identify the neurophysiological mechanisms associated with MAP disorder. However, the electrophysiological effects of MAP+ abusers remain largely unexplored, despite its growing prevalence.

Magnetic resonance imaging (MRI) has become an essential tool in the study of mental illness, which helps clinicians and researchers better understand the patterns of brain activity and structures that are associated with different disorders, such as depression (Wang J. et al., 2022), Alzheimer's disease (Gao et al., 2022a), schizophrenia (Gao et al., 2022b). Few imaging studies, however, have characterized brain dysfunction associated with MAP (Yang et al., 2021; Jia et al., 2022) nor investigated EEG differences in brain dysfunction of MAP. Therefore, neurological dysfunctions related to cognitive performance and psychosis in MA abusers need to be elucidated. The present study aimed to investigate the neurological functions using EEG measurement during addiction Stroop task in MA abusers with or without psychosis compared with age-matched normal participants.

## Materials and methods

### Participants

All participants were recruited from Shenzhen Kangning Hospital and local communities to take part in a set of neuropsychological tests, a psychiatric interview (see Table 1), and electrophysiological recordings. Participants were enrolled into three subgroups: 24 patients with MA-associated psychosis (MAP+, 5 female), 14 MA users without psychosis (MAP-, 2 female), and 31 healthy controls (CTRL, 5 female) with matched age, gender, and education. All participants were required to have normal or corrected-to-normal vision, normal hearing, be aged between 18 and 59 years, and belong to the Chinese Han ethnicity. The MAP+ met a lifetime diagnosis of MA-associated psychosis, while the course of symptoms could be longer than 6 months. The inclusion criteria for the MAP group were 2-fold: (1) Patients had to meet the diagnostic criteria for MA dependence, and they had to exhibit at least three instances of hallucinations and/or delusions; (2) patients were required to have abstained from MA use for a minimum of 15 days to ensure that any observed effects were not due to acute drug use or withdrawal. The MAP- received a diagnosis of MA dependence or abuse, without current or past psychotic symptoms. Subjects were excluded if they had any severe neurological disease, including head trauma, cardiovascular disease, and physical illness. Those with other psychiatric disorders in the DSM-IV axis I, or abuse of other substances, except for tobacco, coffee, and alcohol drinking without alcoholism, were also excluded. Ethical approval for this study was issued by the Research Ethics Committee of Shenzhen Kangning Hospital (2019-k003-01), and written informed consent in accordance with the Declaration of Helsinki was obtained from all the participants after receiving a full explanation of the study. As a token of appreciation for their time spent on the study, each participant was given 200 yuan after completion of the study.

**Table 1:** The demographic data of abstinent methamphetamine users with and without psychosis and healthy control subjects.

	**MAP- (n=13)**	**MAP+ (n=24)**	**CTRL (n=31)**	**χ2 /F/t (p-value)**
**Demographic variables**				
Age, years	38.18 (9.05)	34.19 (5.64)	35.49 (9.26)	1.02 (0.37)
Range	20-53	23-47	20-56	
Female subjects	2	3	5	0.15 (0.93)
Education, years	12.38 (3.57)	11.00 (3.31)	11.97 (3.08)	0.95 (0.39)
**Clinical Variables**				
Methamphetamine use				
Duration, years	2.85 (2.08)	3.83 (2.37)	-	0.25 (0.22)
Range	1-7	1-10	-	
Months abstinent	31.67 (12.79)	33.73 (20.62)	-	5.68 (0.75)
Range	8-54	2-68	-	
Age of first use, years	31.15 (8.76)	27.00 (6.04)	-	1.23 (0.10)
Mean daily use (grams)	0.59 (0.47)	0.60 (0.38)	-	0.26 (0.96)
**Barratt impulsiveness scale**				
Motor	22.31 (6.90)	22.67 (6.458)	-	0.24 (0.88)
Non-planning	27.62 (6.29)	29.54 (8.59)	-	0.90 (0.48)
Attention	26.08 (5.77)	27.62 (6.29)	-	1.68 (0.49)
Total	76.00 (14.42)	80.13 (18.05)	-	1.91 (0.50)

The value represents mean ± SD. MAP- and MAP+ = Methamphetamine without and with psychosis, and CTRL = healthy control, respectively.

p value for the statistical tests: Chi-Square test for gender distribution (across 3 groups), one-way ANOVA for age and education (across 3 groups) and Student two sample t-test (unequal variance assumed) for the drug use measures and Barratt scale (MAP- vs MAP).

### Measures

MA-use patients (including the MAP- and the MAP+) completed the UCLA Natural History Interview (NHI) to provide detailed drug use information, and the Barratt Impulsiveness Scale (BIS-11) (Patton et al., 1995) was used to measure impulsiveness, which consists of 30 items for three domains of impulsivity (attention, motor, and non-planning).

### Addiction stroop task

The addiction Stroop task was constructed and performed on E-Prime 2.0 software (Psychology Software Tools, Inc.). Reaction times and responses for the participant's key presses were recorded. Four stickers were placed on the Q, R, U, and P keys on the keyboard, each representing one of the four colors the participant used to select a response in the Stroop task (red, yellow, blue, and green, respectively). Each word remained on the screen for 1,500 ms or was ended by the reaction button, and each of the 16 words [8 MA-related words and 8 matched control words, which were used in previous research (Haifeng et al., 2015)] were shown in the four colors consequently creating 64 trials for each block, 4 blocks in total. These 256 trials were randomized across participants, and the same category of the words was set not to appear three times consecutively. Before the first block, a practice block would be performed to avoid unfamiliarity with task operations. The fixation cross and the following word were presented on a black background 75 cm away from the eyes. All evaluations were performed following the standardized instructions by trained researchers.

### EEG acquisition and processing

While participants performed the Stroop task, EEG data were recorded from 32 Ag/AgCl scalp electrodes (BrainCap, GmbH, Germany) according to the international 10–20 system. The placement of the recording reference was at Cz, while the ground was positioned at approximately AFz. The impedances were kept below 10 kΩ with the sampling frequency at 1,000 Hz.

Utilizing MATLAB (MathWorks, Natick, MA, United States) and the EEGLAB toolbox (Delorme and Makeig, 2004), we processed the continuous EEG data with in-house scripts. An offline digital band-pass filter (0.1–30 Hz) was applied. Epochs were extracted from −200 to 1,000 ms relative to the onset of the word stimulus and baseline corrected using the prestimulus interval (−200 to 0 ms). Independent component analysis (ICA) was used to correct eye movement, muscle artifacts, and heartbeat artifacts. All EEG epochs were processed for artifact detection by visual inspection and EEGLAB, and detection of obvious eye blinks and epochs with amplitude values exceeding ±100 mV at any electrode were rejected and later re-referenced to the average reference (Tafuro et al., 2019; Overbye et al., 2021). To guarantee the quality of data, patients with >20% of bad epochs for each condition and/or five bad channels were removed from the analysis, and one MAP- participant with more than 20% of bad epochs was excluded.

### ERPs

ERP analyses mainly focused on the components of N200, P300, and N450. Time course of the average of left-anterior frontal channels was used to obtain the amplitude and latency of N200, P300, and N450. The time windows for evaluating ERP peaks were determined by inspecting the grand-averaged waveforms; the time windows are as follows: 200 to 300 ms for N200; 250 to 450 ms for P300; and 400 to 650 ms for N450. The mean amplitude and latency (FDR corrected) of these components in MA-related word trials were measured on left-anterior electrodes (F3, F7, FC5 electrodes), and a 50% fractional area technique (Kiesel et al., 2008) was applied to measure the latency of components.

### Statistics

For demographic and clinical characteristics, the groups were compared with Student's *t*-test or one-way ANOVAs (analysis of variance) followed by Tukey's *post-hoc* test, and a chi-square test was conducted for categorical variable comparisons (Table 1). For the Stroop effect, error rate and mean reaction time (RT) were analyzed using a 3 × 2 mixed-design ANOVA with the groups (MAP- vs. MAP+ vs. HCs) as a between-subjects factor and stimulus type (MA word vs. neutral word) as a within-subject factor. In addition, only MA word trials were considered in the EEG data analysis; a one-way analysis of covariance (ANCOVA) followed by Tukey's *post-hoc* test was used to analyze the amplitude and latency of the groups in outcome measures, with the groups as the between-subject factor. The entire statistical analysis was conducted using IBM SPSS Statistics version 26 (IBM Corp., Armonk, N.Y., USA).

## Results

### Participant characteristics and behavior

The outcomes (mean values) of all dependent variables (CTRL, *n* =31; MAP-, *n* = 13; MAP+, *n* = 24) are presented in Table 1. There was no significant difference in the mean age and education between groups. MAP+ and MAP- show no difference in methamphetamine use variables and Barratt impulsiveness scores.

**Figure 1:**
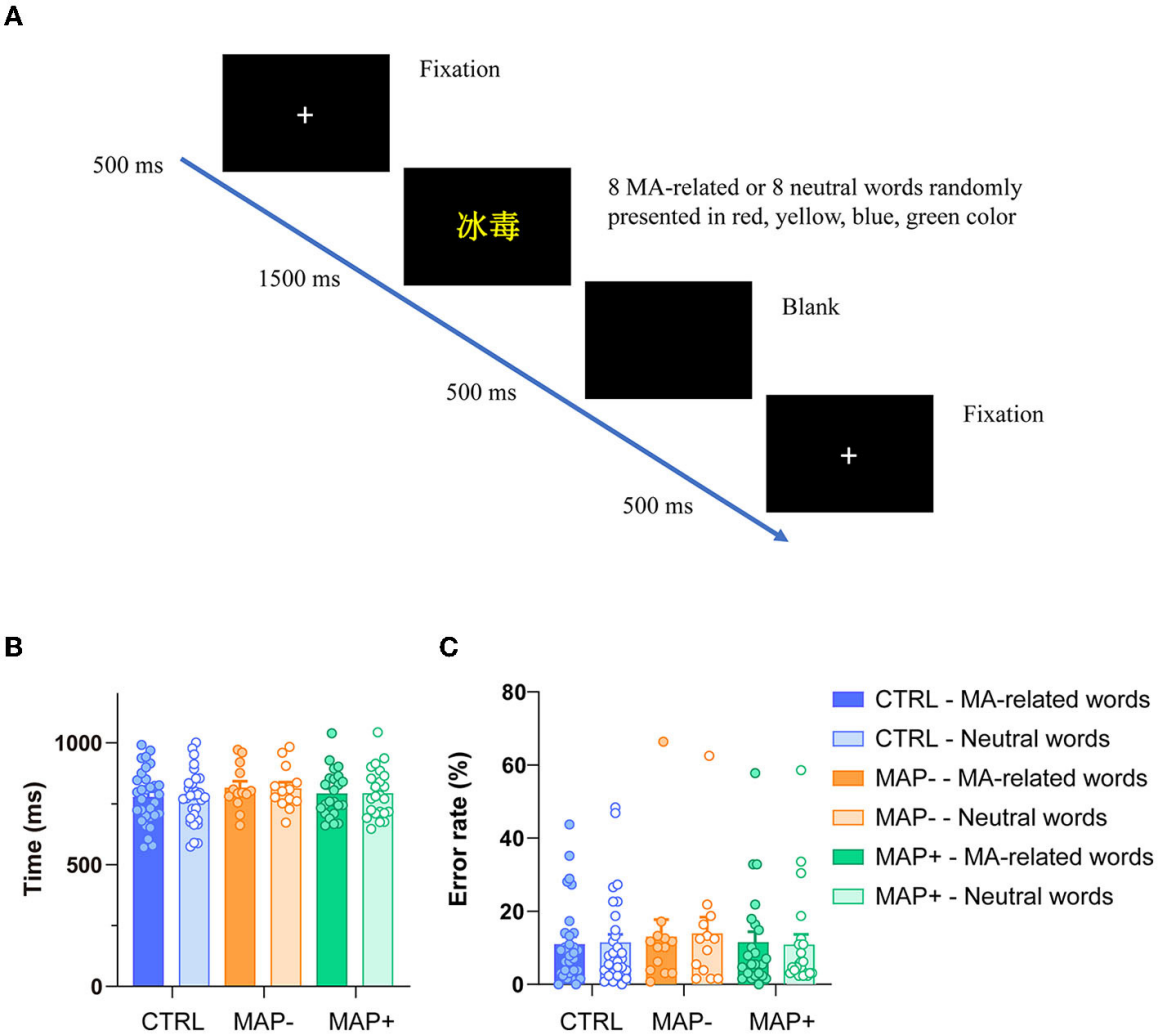
Illustration of the experimental procedure and behavioral performance. **(A)** Sequence of trial events in an MA-related words trial. **(B)** RT for correct trial across conditions. **(C)** The error rate committed on each group and each trial type.

The descriptive behavioral data for both groups, including mean RTs (ms) and error rate (%) for each condition, are shown in Figure 1. The two-way ANOVAs were conducted on the mean RTs and the error rates. For the analysis of the RTs, all incorrect trials were excluded. Figure 1A illustrates the procedure of the MA addiction Stroop task. A 3 × 2 ANOVA on the RTs showed no significant main effect for group or condition (MA-related words and neutral words), respectively (Figure 1B, group: F (2, 128) = 1.270, *p* = 0.284; condition: F (1, 128) = 0.008, *p* = 0.930), and the group × condition interaction was also not significant (Figure 1B, F (2, 128) = 0.007, *p* = 0.993). There were no significant differences between the groups in the error rate of each condition (Figure 1C, group: F (2, 128) = 0.311, *p* = 0.733; condition: F (1, 128) = 0.008, *p* = 0.930; group × condition: F (2, 128) = 0.031, *p* = 0.970).

### ERPs

**Figure 2:**
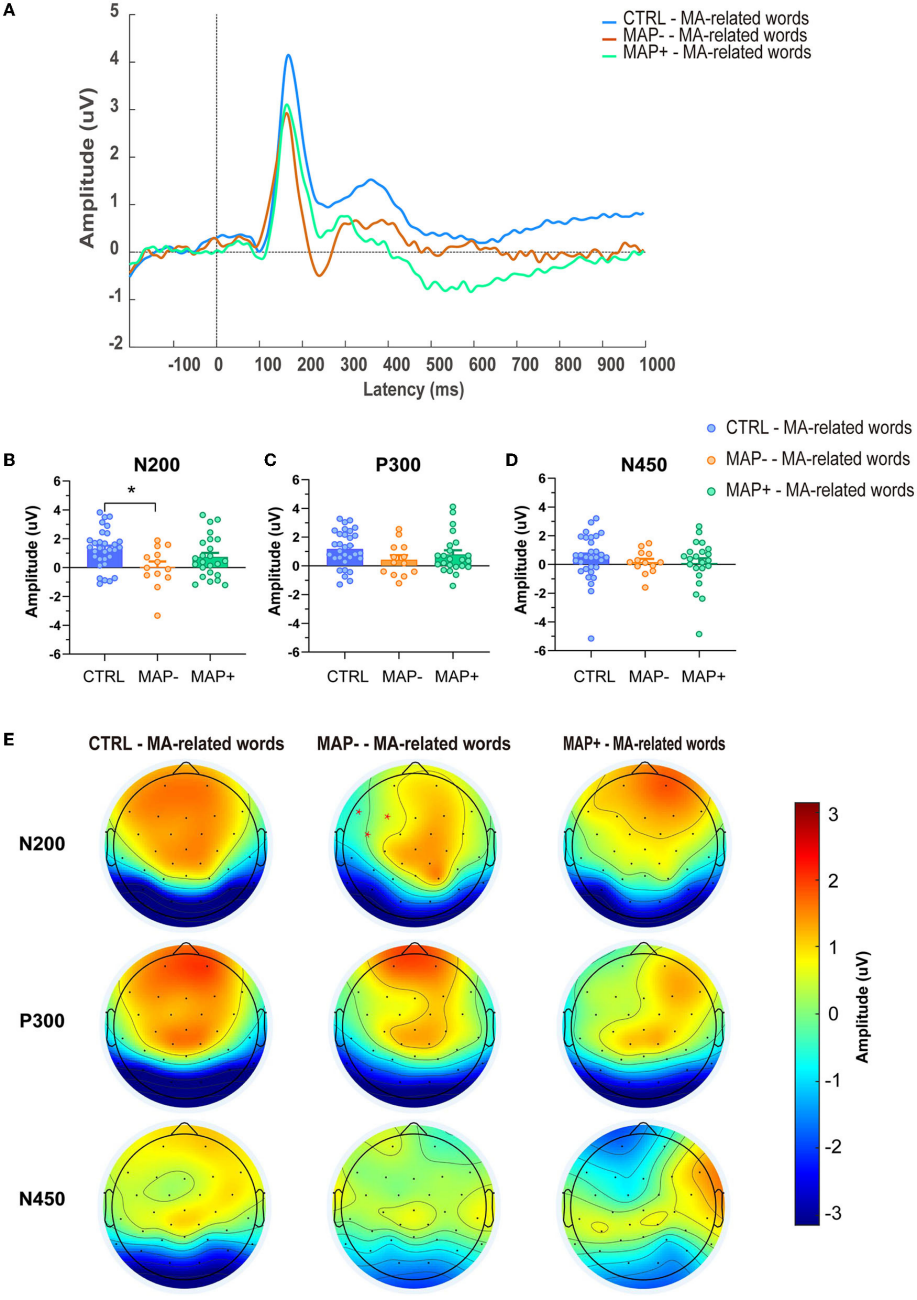
Averaged ERP amplitudes and topographical maps. **(A)** Grand mean averages (uV) of ERP waveforms to the MA-related words in the CTRL, MAP-, MAP+ groups across left-anterior electrode sites. **(B–D)** Mean N200, P300, and N450 amplitudes (uV) in each group, averaged across left-anterior electrode sites. Error bars represent SEMs. **(E)** Topographical maps of N200, P30, and N450 across different groups.

The N200, P300, N450 peaks were observed only in MA-related word condition of each group. The ERP waveforms and topographical distributions of the N200, P300, and N450 components for the analyzed trial types are shown in Figures 2A, E. A significant interaction emerged for N200 amplitude between the groups (Figure 2B, F_2.65_ = 3, *p* = 0.028); subsequently, the *post-hoc* analysis showed that compared with the CTRL group, the MAP- group showed a more negative N200 amplitude in the MA-related word condition on left-anterior electrodes (*p* = 0.009), while no significant effects emerged in the mean amplitude of P300 (Figure 2C, F (2.64) = 1.668, *p* = 0.196) and N450 (Figure 2D, F (2.64) = 0.455, *p* = 0.636). With regard to latency, none of the changes in the latency of N200, P300, and N450 were statistically significant (please refer to Supplementary Figures 1A–C, N200: F (2.65) = 1.023, *p* = 0.365; P300: F (2.65) = 0.177, *p* = 0.838; N450: F (2.65) = 0.039, *p* = 0.962, respectively).

### Correlations with Barratt impulsiveness scores

**Figure 3:**
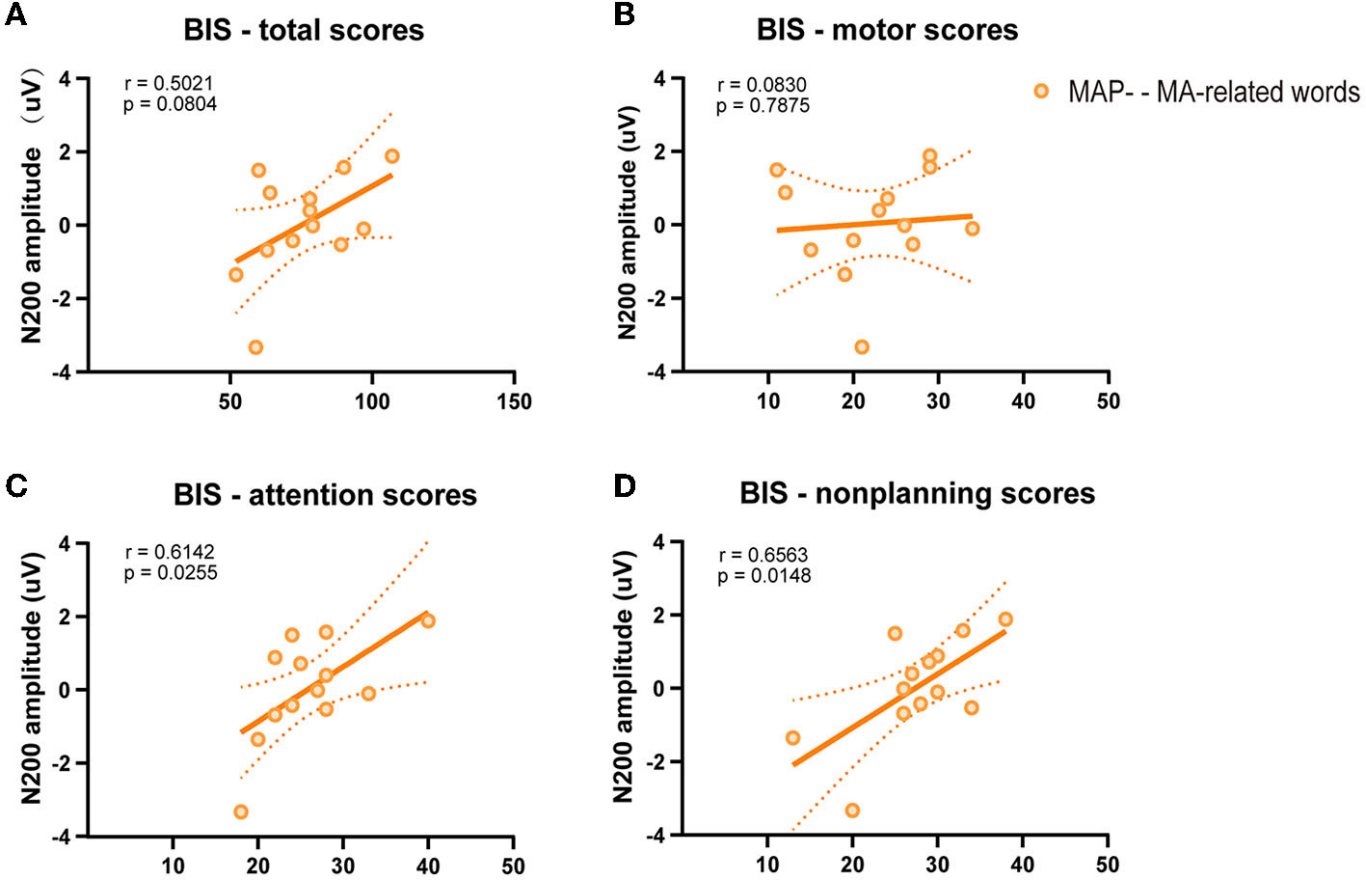
Scatterplot of the relation between N200 amplitude and Barratt impulsiveness scores. **(A)** Barratt total scores. **(B)** Barratt motor scores. **(C)** Barratt attentional scores. **(D)** Barratt non-planning scores.

To further examine the relationship between behavior and electrophysiological signature, we correlated the Barratt total score and sub-domain scores separately with the difference in amplitudes of the N200 on MA-related word trials. Pearson's correlation test was used to assess the correlation between variables (Figures 3A–D). A positive relationship of N200 amplitude with attentional impulsivity score (*p* = 0.026, *r* = 0.614) and non-planning impulsivity score (*p* = 0.015, *r* = 0.656) was observed in MAP- abusers, while this positive correlation was not found in Barratt total score (*p* = 0.080, *r* = 0.502) and motor impulsivity score (*p* = 0.788, *r* = 0.083).

## Discussion

This is an initial study to examine the relationships between ERP time course and performance on an addiction Stroop task in MA abusers with or without psychosis history. The current study investigated whether MA abusers with or without a psychosis history are characterized by deficits in ERPs during the addiction Stroop task. We found that MA-related words stimulus elicited a more negative N200 amplitude over left-anterior electrodes in MAP- abusers; furthermore, a positive association between N200 amplitude and Barratt attentional scores and non-planning scores was observed, while no such differences were found in MAP+ abusers.

Attentional bias toward addiction-related cues can impede executive functions that are pivotal in sustaining abstinence from drugs. To measure this attention bias, addiction Stroop tasks, which are similar to the classical Stroop tasks but comprised of both drug-related and neutral words, are used when assessing research participants who use drugs. Despite the lack of any noteworthy disparity in behavioral performance data between groups, high-temporal-resolution ERP technology is more adept at detecting the subtle distinctions in attentional processes, as more negative amplitudes over anterior electrode electrodes elicited by MA-related words that were observed among MAP- abusers and not MAP+ abusers can be attributed to the attentional bias of MAP abusers for MA-related cues. Previous studies (Potvin et al., 2018; Guerin et al., 2019) have shown that those with methamphetamine use disorder have cognitive deficits in many areas compared with controls, with inhibitory control, assessed through the color-word Stroop task, being particularly impaired. Minor addiction Stroop effect can be attributed to certain factors. First, the duration of withdrawal time may affect the Stroop effect, as MA abusers in our study were abstinent for a long time ranging from 2 to 68 months. In a recent study, it was observed that individuals who had recently abstained from MA abuse displayed a higher level of Stroop RT interference in comparison with both the control group and those who had been abstinent from MA abuse for a longer period of time. Conversely, no significant difference was observed between the long-term abstinent MA-abusing individuals and the control group (Salo et al., 2009). Second, RTs and error rate are not sensitive indicators of attentional bias from MA abusers in the Stroop task. Previous studies had showed no difference in RTs and error rate, however, there was evidence of intraindividual variability (IIV) and excessively long RTs (tau) in MA abusers who were abstinent for 2 to 60 months (Fassbender et al., 2015). Finally, quiet investigations of the addiction Stroop task did not detect any behavioral performance between drug abusers and participants, with quick response and high accuracy (Fehr et al., 2006; Haifeng et al., 2015; Chen et al., 2021), which might refer to the ceiling effect of this task, so a task of low difficulty would not be able to distinguish between drug users and the general population in terms of behavior.

ERPs, a highly informative and dynamic method of tracking brain activity with a high temporal resolution, are characterized by a series of positive and negative components. N200, P300, N450, and conflict slow potential (SP) latency and amplitude on the variable Stroop test were usually measured to differ between conditions (Ergen et al., 2014; Sahinoglu and Dogan, 2016; Fang et al., 2022). According to a prior investigation, smoking-associated images elicited a relatively negative response at frontal and central electrode locations during the 200–250 ms timeframe, as well as left frontal negativity between 400 and 500 ms at the F7 site, as compared to neutral images in the context of the addiction Stroop test (Fehr et al., 2007). Although we found relative negativity at left-anterior electrode sites between 200 and 300 ms, N450 did not reach a significant level. The functional implications of N450 have been extensively studied in order to differentiate between conflict resolution and response selection processes. This has been achieved by analyzing the ERP data obtained from various versions of the Stroop test. The results indicate that N450 exhibits greater negativity in response to incongruent trials as compared to congruent trials (Chuderski et al., 2016; Guo et al., 2018). Further inquiry into the part that N450 plays in addiction is needed in the future. Additionally, an ERP study of the addiction Stroop task revealed that abstinence from MA resulted in a decrease in left-anterior P300 to MA-related words at 3 and 6 months, and this decrease was associated with a decrease in craving (Haifeng et al., 2015). Despite the lack of P300 alteration in MA abusers in the present study, it is reasonable to assume that the period of abstinence is a crucial factor in MA-related words related to P300. It is apparent that further EEG investigations on the addiction Stroop task necessitate validation in subsequent research.

Correlations between ERPs and measured results provided useful information and may further multiply the results of the present research. Pearson's correlation analysis between the Barratt scores and the N200 amplitude was conducted to determine the relationship between subjective traits and N200 amplitude elicited by MA-related cues. A positive association between N200 amplitude and Barratt attentional scores and non-planning scores was observed in MAP- abusers. It may be concluded that those people who scored high in the Barratt attentional score and non-planning score showed a higher N200 amplitude in MA-related word condition.

## Data availability statement

The original contributions presented in the study are included in the article/Supplementary material, further inquiries can be directed to the corresponding authors.

## Ethics statement

The studies involving human participants were reviewed and approved by Research Ethics Committee of Shenzhen Kangning Hospital. The patients/participants provided their written informed consent to participate in this study.

## Author contributions

MY and XJ were instrumental in the conception and design of the work. FL and ZK were involved in the acquisition of the data. YL, YZ, and GY were involved in the investigation and formal analysis. GH, CH, and JY were responsible for analyzing and interpreting the data and as well as writing the article. In the course of a rigorous review process, GH, IJ, and CJ collaborated to scrutinize the article and have committed to being answerable for all elements of the project. All authors contributed to the article and approved the submitted version.

## Supplementary material

Supplementary Figure 1 Averaged ERP latencies. **(A–C)** Mean N200, P300, and N450 amplitudes (uV) in each group, averaged across left-anterior electrode sites, respectively. Error bars represent SEMs.
