# Supplementary figures and images for: Neural basis of the attention bias during addiction stroop task in methamphetamine-dependent patients with and without a history of psychosis: an ERP study
Source: Front Psychol. 2023 Jun 9;14:1173711. doi: 10.3389/fpsyg.2023.1173711 (PMC10288148; doi:10.3389/fpsyg.2023.1173711)

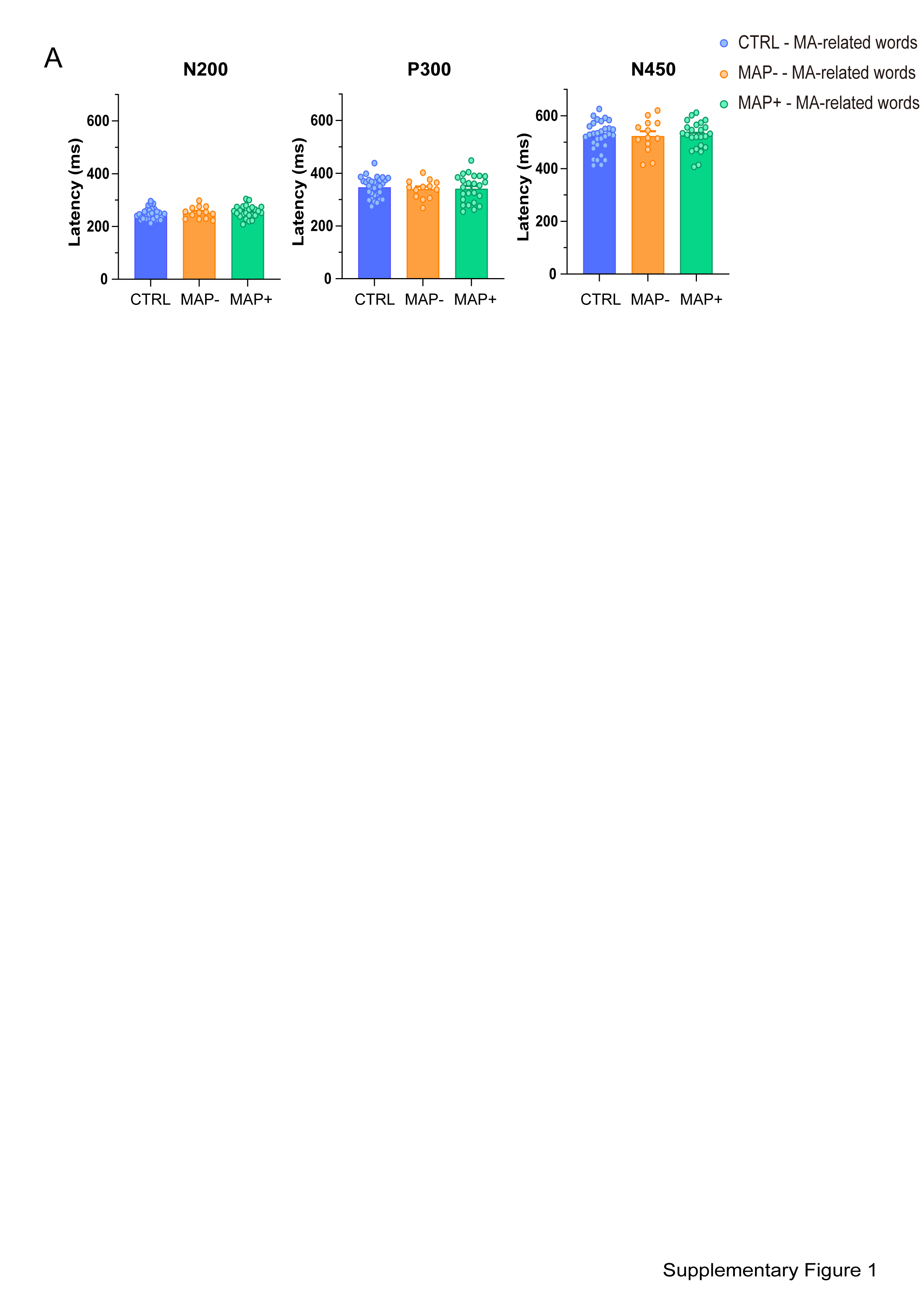

Supplement: Supplementary Figure 1 — Averaged ERP latencies. (A–C) Mean N200, P300, and N450 amplitudes (uV) in each group, averaged across left-anterior electrode sites, respectively. Error bars represent SEMs. [file Image_1.TIF]
